# 2-Methyl-3,5-dinitro­benzoic acid

**DOI:** 10.1107/S1600536809042627

**Published:** 2009-10-23

**Authors:** M. Nawaz Tahir, Abdul Rauf Raza, Aisha Saddiqa, Muhammad Danish, Iram Saleem

**Affiliations:** a Department of Physics, University of Sargodha, Sargodha, Pakistan; b Department of Chemistry, University of Sargodha, Sargodha, Pakistan

## Abstract

In the title compound, C_8_H_6_N_2_O_6_, the O atoms of the nitro groups, the methyl H atoms and the carboxyl C=O and C—OH groups are disordered over two sets of sites with an occupancy ratio of 0.595 (16):0.405 (16). In the crystal, inversion dimers linked by pairs of O—H⋯O hydrogen bonds arise for both carboxyl disorder components and C—H⋯O bonds and weak C—H⋯π inter­actions consolidate the packing.

## Related literature

For general background to isocoumarins, see: Hill (1986 ▶); Varanda *et al.* (2004 ▶). For related structures, see: Prince *et al.* (1991 ▶); Sarma & Nagaraju (2000 ▶).
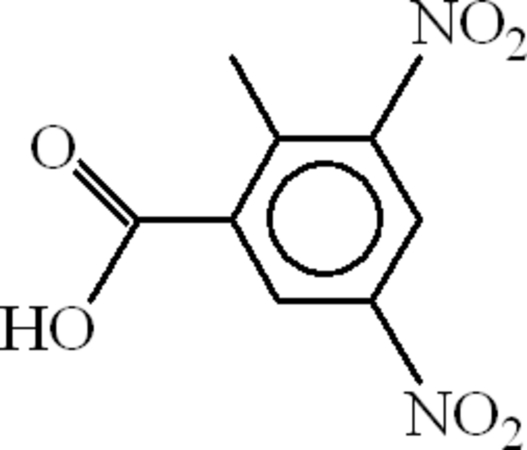


## Experimental

### 

#### Crystal data

C_8_H_6_N_2_O_6_
*M* *_r_* = 226.15
Monoclinic, 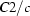

*a* = 26.8441 (16) Å
*b* = 5.1044 (3) Å
*c* = 13.8853 (10) Å
β = 104.544 (3)°
*V* = 1841.6 (2) Å^3^
*Z* = 8
Mo *K*α radiation
μ = 0.14 mm^−1^
*T* = 296 K
0.28 × 0.09 × 0.08 mm

#### Data collection

Bruker Kappa APEXII CCD diffractometer
Absorption correction: multi-scan (*SADABS*; Bruker, 2005 ▶) *T*_min_ = 0.985, *T*_max_ = 0.987
8618 measured reflections
2019 independent reflections
1626 reflections with *I* > 2σ(*I*)
*R*_int_ = 0.025

#### Refinement

*R*[*F* ^2^ > 2σ(*F* ^2^)] = 0.035
*wR*(*F* ^2^) = 0.098
*S* = 1.07
2019 reflections
189 parameters
H atoms treated by a mixture of independent and constrained refinement
Δρ_max_ = 0.22 e Å^−3^
Δρ_min_ = −0.17 e Å^−3^

### 

Data collection: *APEX2* (Bruker, 2007 ▶); cell refinement: *SAINT* (Bruker, 2007 ▶); data reduction: *SAINT*; program(s) used to solve structure: *SHELXS97* (Sheldrick, 2008 ▶); program(s) used to refine structure: *SHELXL97* (Sheldrick, 2008 ▶); molecular graphics: *ORTEP-3 for Windows* (Farrugia, 1997 ▶) and *PLATON* (Spek, 2009 ▶); software used to prepare material for publication: *WinGX* (Farrugia, 1999 ▶) and *PLATON*.

## Supplementary Material

Crystal structure: contains datablocks global, I. DOI: 10.1107/S1600536809042627/hb5136sup1.cif

Structure factors: contains datablocks I. DOI: 10.1107/S1600536809042627/hb5136Isup2.hkl

Additional supplementary materials:  crystallographic information; 3D view; checkCIF report

## Figures and Tables

**Table 1:** Hydrogen-bond geometry (Å, °)

*D*—H⋯*A*	*D*—H	H⋯*A*	*D*⋯*A*	*D*—H⋯*A*
O1—H1⋯O2^i^	0.83 (4)	1.80 (4)	2.6216 (16)	175 (3)
C8—H8*B*⋯O5*A*^ii^	0.96	2.55	3.385 (11)	145
C8—H8*C*⋯O3*A*	0.96	2.43	3.023 (11)	120
C8—H8*A*⋯*Cg*1^iii^	0.96	2.96	3.781 (2)	144
C8—H8*E*⋯*Cg*1^iii^	0.96	2.96	3.781 (2)	144

Symmetry codes: (i) 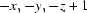; (ii) 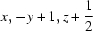; (iii) 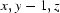. *Cg*1 is the centroid of the C1–C6 benzene ring.

**Fig. 1.**
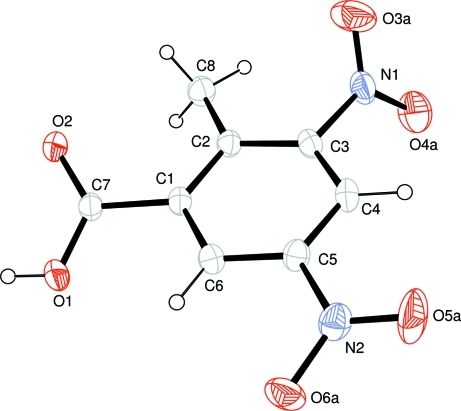
View of (I) with the atom numbering scheme having atoms of greater occupancy ratio. The displacement ellipsoids are drawn at the 30% probability level. H-atoms are shown by small circles of arbitrary radii.

**Fig. 2.**
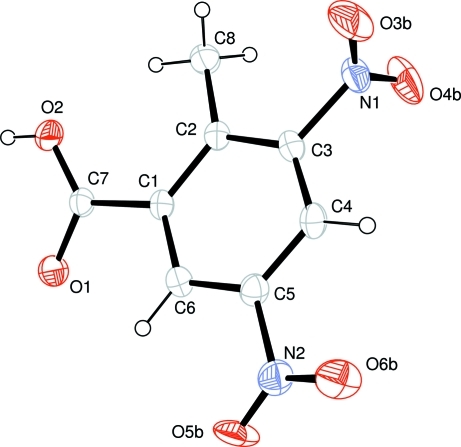
View of (I) with the atom numbering scheme having atoms of smaller occupancy ratio. The displacement ellipsoids are drawn at the 30% probability level. H-atoms are shown by small circles of arbitrary radii.

**Fig. 3.**
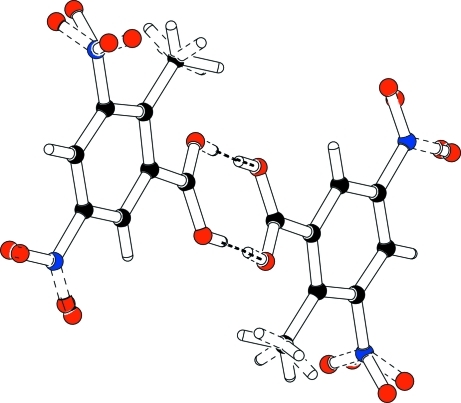
The partial packing of (I), which shows that molecules form inversion dimers.

## supplementary crystallographic information

### Comment

Isocoumarins are secondary metabolites, derived from acetate pathway, which are structurally related to coumarins but with inverted lactone ring. Isocoumarins shows a wide range of applications and biological activities including anti-cancer (Varanda *et al.*, 2004), anti-tumor (Hill *et al.*, 1986) *etc*.

The title compound (I, Fig. 1) is an intermediate towards the synthesis of substituted homophthallic acid that is a precursor for the synthesis of isocoumarins.

The ctystal structures of (II) 2,4-Dinitrotoluene (Sarma & Nagaraju 2000), (III) 3,5-Dinitrobenzoic acid (Prince *et al.*, 1991) have been reported. The title compound contains both of these moieties.

The O-atoms of nito groups are disordered over two sets of sites with occupancy ratio of 0.595 (16):0.405 (16). Due to this disorder the H-atoms of CH_3_ and OH groups are also disordered with same occupancy ratio. The title compound consist of conventional carboxylate dimers (Fig. 2). The benzene ring A (C1–C6) and carboxyl group B (O1/C7/O2) are oriented at a dihedral angle of 23.82 (15)°. The disordered nitro groups C (O3A/N1/O4A), D (O3B/N1/O4B), E (O5A/N2/O6A) and F (O5B/N1/O6B) are certainly planar. The values of dihedral angles for C/E and D/F are 57 (1) and 76 (1)°, respectively. The molecules are stabilized due to H-bondings and C—H···π interactions (Table 1).

### Experimental

HNO_3_ (28.0 g, 0.7 mol) was added as drops to an ice-chilled (273 K) solution of *o*-toluic acid (13.6 g, 0.1 mol) in H_2_SO_4_ (110.4 g, 11.2 mol) with constant stirring. The reaction mixture was stirred for 15 minutes, left overnight on stirring at room temperature and then refluxed at 373 K for 4 h. More HNO_3_ (21.0 g, 0.69 mol) was added after cooling to room temperature and refluxed for further 3 h. The reaction mixture was cooled to room temperature and poured to ice. The precipitates were filtered, washed with distilled water to remove free sulfates and nitrates. Recrystallization from methanol/water (1:1) afforded yellow needles of (I) suitable for *x*-ray diffraction. Yield 92%.

### Refinement

The O-atoms of NO_2_ groups along with H-atoms of CH_3_ and OH groups are disordered. The coordinates of H-atoms of hydroxy group were refined.

H-atoms were positioned geometrically, with C—H = 0.93 and 0.96 Å for aryl and methyl H, respectively and constrained to ride on their parent atoms, with *U*_iso_(H) = *xU*_eq_(C, O), where *x* = 1.5 for methyl and 1.2 for all other H atoms.

### Crystal data

**Table:**

C_8_H_6_N_2_O_6_	*F*(000) = 928
*M**_r_* = 226.15	*D*_x_ = 1.631 Mg m^−^^3^
Monoclinic, *C*2/*c*	Mo *K*α radiation, λ = 0.71073 Å
Hall symbol: -C 2yc	Cell parameters from 2019 reflections
*a* = 26.8441 (16) Å	θ = 3.0–27.1°
*b* = 5.1044 (3) Å	µ = 0.14 mm^−^^1^
*c* = 13.8853 (10) Å	*T* = 296 K
β = 104.544 (3)°	Needle, yellow
*V* = 1841.6 (2) Å^3^	0.28 × 0.09 × 0.08 mm
*Z* = 8	

### Data collection

**Table:**

Bruker Kappa APEXII CCD diffractometer	2019 independent reflections
Radiation source: fine-focus sealed tube	1626 reflections with *I* > 2σ(*I*)
graphite	*R*_int_ = 0.025
Detector resolution: 7.60 pixels mm^-1^	θ_max_ = 27.1°, θ_min_ = 3.0°
ω scans	*h* = −34→21
Absorption correction: multi-scan (*SADABS*; Bruker, 2005)	*k* = −6→6
*T*_min_ = 0.985, *T*_max_ = 0.987	*l* = −16→17
8618 measured reflections	

### Refinement

**Table:**

Refinement on *F*^2^	Primary atom site location: structure-invariant direct methods
Least-squares matrix: full	Secondary atom site location: difference Fourier map
*R*[*F*^2^ > 2σ(*F*^2^)] = 0.035	Hydrogen site location: inferred from neighbouring sites
*wR*(*F*^2^) = 0.098	H atoms treated by a mixture of independent and constrained refinement
*S* = 1.07	*w* = 1/[σ^2^(*F*_o_^2^) + (0.0466*P*)^2^ + 0.7672*P*] where *P* = (*F*_o_^2^ + 2*F*_c_^2^)/3
2019 reflections	(Δ/σ)_max_ < 0.001
189 parameters	Δρ_max_ = 0.22 e Å^−^^3^
0 restraints	Δρ_min_ = −0.17 e Å^−^^3^

### Special details

**Table:**

Geometry. Bond distances, angles *etc*. have been calculated using the rounded fractional coordinates. All su's are estimated from the variances of the (full) variance-covariance matrix. The cell e.s.d.'s are taken into account in the estimation of distances, angles and torsion angles
Refinement. Refinement of *F*^2^ against ALL reflections. The weighted *R*-factor *wR* and goodness of fit *S* are based on *F*^2^, conventional *R*-factors *R* are based on *F*, with *F* set to zero for negative *F*^2^. The threshold expression of *F*^2^ > σ(*F*^2^) is used only for calculating *R*-factors(gt) *etc*. and is not relevant to the choice of reflections for refinement. *R*-factors based on *F*^2^ are statistically about twice as large as those based on *F*, and *R*- factors based on ALL data will be even larger.

### Fractional atomic coordinates and isotropic or equivalent isotropic displacement parameters (Å^2^)

**Table:**

	*x*	*y*	*z*	*U*_iso_*/*U*_eq_	Occ. (<1)
O1	0.01701 (4)	0.2581 (2)	0.43564 (8)	0.0512 (3)	
O2	0.05404 (4)	−0.13177 (19)	0.47311 (8)	0.0478 (3)	
O3A	0.2288 (4)	−0.200 (2)	0.3383 (8)	0.083 (2)	0.595 (16)
O4A	0.2604 (2)	0.1735 (16)	0.3731 (8)	0.0879 (18)	0.595 (16)
O5A	0.1204 (4)	0.696 (2)	0.1252 (9)	0.082 (3)	0.595 (16)
O6A	0.0510 (4)	0.781 (2)	0.1802 (7)	0.0573 (14)	0.595 (16)
N1	0.22362 (4)	0.0323 (3)	0.35244 (10)	0.0497 (4)	
N2	0.09302 (5)	0.6426 (3)	0.17734 (9)	0.0485 (4)	
C1	0.09152 (4)	0.1704 (2)	0.38137 (9)	0.0325 (3)	
C2	0.14060 (4)	0.0536 (2)	0.39970 (9)	0.0335 (3)	
C3	0.17128 (4)	0.1421 (3)	0.33837 (10)	0.0367 (4)	
C4	0.15720 (5)	0.3269 (3)	0.26482 (10)	0.0399 (4)	
C5	0.10921 (5)	0.4375 (3)	0.25252 (9)	0.0371 (4)	
C6	0.07666 (4)	0.3649 (3)	0.31037 (9)	0.0360 (4)	
C7	0.05193 (4)	0.0909 (3)	0.43530 (9)	0.0346 (4)	
C8	0.16097 (5)	−0.1411 (3)	0.48095 (11)	0.0448 (4)	
O4B	0.2566 (3)	0.149 (3)	0.4132 (8)	0.084 (3)	0.405 (16)
O5B	0.0598 (5)	0.750 (3)	0.1754 (11)	0.061 (2)	0.405 (16)
O6B	0.1179 (7)	0.655 (3)	0.1105 (11)	0.061 (2)	0.405 (16)
O3B	0.2269 (6)	−0.152 (3)	0.3038 (13)	0.094 (4)	0.405 (16)
H8B	0.15619	−0.07488	0.54272	0.0672*	0.595 (16)
H8C	0.19697	−0.16935	0.48691	0.0672*	0.595 (16)
H8A	0.14278	−0.30373	0.46522	0.0672*	0.595 (16)
H1	−0.0046 (14)	0.209 (6)	0.465 (2)	0.0614*	0.595 (16)
H4	0.17896	0.37504	0.22525	0.0479*	
H6	0.04484	0.44612	0.30177	0.0432*	
H2	0.0315 (19)	−0.169 (9)	0.498 (4)	0.0614*	0.405 (16)
H8D	0.19134	−0.07189	0.52575	0.0672*	0.405 (16)
H8E	0.16930	−0.30148	0.45242	0.0672*	0.405 (16)
H8F	0.13531	−0.17459	0.51669	0.0672*	0.405 (16)

### Atomic displacement parameters (Å^2^)

**Table:**

	*U*^11^	*U*^22^	*U*^33^	*U*^12^	*U*^13^	*U*^23^
O1	0.0444 (5)	0.0495 (6)	0.0706 (7)	0.0066 (4)	0.0349 (5)	0.0127 (5)
O2	0.0450 (5)	0.0407 (5)	0.0658 (7)	−0.0019 (4)	0.0293 (5)	0.0092 (5)
O3A	0.058 (2)	0.064 (3)	0.121 (5)	0.0211 (19)	0.012 (3)	−0.014 (3)
O4A	0.0343 (13)	0.091 (2)	0.142 (5)	−0.0100 (13)	0.029 (3)	−0.005 (3)
O5A	0.068 (3)	0.114 (5)	0.071 (5)	−0.004 (3)	0.031 (3)	0.051 (4)
O6A	0.052 (3)	0.060 (2)	0.061 (2)	0.027 (2)	0.016 (2)	0.0131 (16)
N1	0.0356 (6)	0.0591 (8)	0.0593 (8)	0.0035 (6)	0.0213 (6)	0.0073 (7)
N2	0.0488 (7)	0.0541 (7)	0.0416 (7)	−0.0018 (6)	0.0093 (6)	0.0104 (6)
C1	0.0303 (6)	0.0364 (6)	0.0325 (6)	−0.0048 (5)	0.0109 (5)	−0.0019 (5)
C2	0.0315 (6)	0.0354 (6)	0.0345 (6)	−0.0036 (5)	0.0100 (5)	−0.0024 (5)
C3	0.0295 (6)	0.0424 (7)	0.0401 (7)	−0.0005 (5)	0.0124 (5)	−0.0016 (6)
C4	0.0364 (6)	0.0489 (8)	0.0388 (7)	−0.0063 (6)	0.0175 (5)	0.0012 (6)
C5	0.0374 (6)	0.0421 (7)	0.0320 (6)	−0.0034 (5)	0.0089 (5)	0.0039 (5)
C6	0.0300 (6)	0.0414 (7)	0.0368 (7)	−0.0013 (5)	0.0090 (5)	−0.0003 (5)
C7	0.0316 (6)	0.0369 (6)	0.0376 (7)	−0.0031 (5)	0.0128 (5)	−0.0007 (5)
C8	0.0416 (7)	0.0468 (8)	0.0466 (8)	0.0033 (6)	0.0124 (6)	0.0089 (6)
O4B	0.029 (3)	0.115 (5)	0.102 (5)	0.000 (3)	0.005 (3)	−0.011 (4)
O5B	0.064 (5)	0.068 (4)	0.064 (3)	0.046 (3)	0.039 (3)	0.032 (3)
O6B	0.075 (5)	0.075 (3)	0.040 (2)	0.020 (3)	0.027 (2)	0.017 (2)
O3B	0.070 (5)	0.074 (6)	0.152 (10)	0.020 (3)	0.053 (6)	−0.025 (6)

### Geometric parameters (Å, °)

**Table:**

O1—C7	1.2686 (17)	C1—C6	1.3854 (18)
O2—C7	1.2473 (18)	C1—C7	1.5015 (16)
O3A—N1	1.216 (10)	C2—C3	1.3999 (17)
O3B—N1	1.174 (16)	C2—C8	1.4999 (19)
O4A—N1	1.198 (7)	C3—C4	1.372 (2)
O4B—N1	1.215 (12)	C4—C5	1.377 (2)
O5A—N2	1.186 (12)	C5—C6	1.3779 (18)
O5B—N2	1.041 (14)	C4—H4	0.9300
O6A—N2	1.340 (11)	C6—H6	0.9300
O6B—N2	1.274 (17)	C8—H8A	0.9600
O1—H1	0.83 (4)	C8—H8B	0.9600
O2—H2	0.79 (5)	C8—H8C	0.9600
N1—C3	1.4794 (17)	C8—H8D	0.9600
N2—C5	1.465 (2)	C8—H8E	0.9600
C1—C2	1.4098 (16)	C8—H8F	0.9600
			
C7—O1—H1	114 (2)	C4—C5—C6	121.86 (13)
C7—O2—H2	116 (3)	N2—C5—C4	118.82 (12)
O4A—N1—C3	120.1 (4)	N2—C5—C6	119.31 (12)
O3B—N1—C3	115.7 (8)	C1—C6—C5	119.75 (11)
O3A—N1—C3	119.5 (5)	O2—C7—C1	119.55 (11)
O3B—N1—O4B	130.2 (10)	O1—C7—O2	124.54 (12)
O4B—N1—C3	114.1 (6)	O1—C7—C1	115.86 (12)
O3A—N1—O4A	120.3 (6)	C3—C4—H4	122.00
O5A—N2—O6A	123.5 (7)	C5—C4—H4	122.00
O5B—N2—C5	119.6 (8)	C1—C6—H6	120.00
O6B—N2—C5	116.0 (7)	C5—C6—H6	120.00
O5B—N2—O6B	123.9 (11)	C2—C8—H8A	109.00
O6A—N2—C5	117.2 (4)	C2—C8—H8B	109.00
O5A—N2—C5	118.7 (5)	C2—C8—H8C	109.00
C2—C1—C7	122.82 (10)	C2—C8—H8D	109.00
C2—C1—C6	121.37 (10)	C2—C8—H8E	109.00
C6—C1—C7	115.80 (10)	C2—C8—H8F	109.00
C3—C2—C8	120.82 (11)	H8A—C8—H8B	109.00
C1—C2—C8	124.31 (11)	H8A—C8—H8C	109.00
C1—C2—C3	114.81 (11)	H8B—C8—H8C	109.00
N1—C3—C2	118.80 (12)	H8D—C8—H8E	109.00
N1—C3—C4	115.71 (12)	H8D—C8—H8F	109.00
C2—C3—C4	125.49 (12)	H8E—C8—H8F	109.00
C3—C4—C5	116.64 (12)		
			
O3A—N1—C3—C2	63.2 (6)	C2—C1—C7—O1	158.86 (11)
O3A—N1—C3—C4	−117.2 (6)	C2—C1—C7—O2	−23.53 (18)
O4A—N1—C3—C2	−120.3 (6)	C6—C1—C7—O1	−22.16 (17)
O4A—N1—C3—C4	59.4 (6)	C6—C1—C7—O2	155.46 (12)
O5A—N2—C5—C4	4.6 (6)	C1—C2—C3—N1	179.58 (12)
O5A—N2—C5—C6	−176.9 (6)	C1—C2—C3—C4	−0.1 (2)
O6A—N2—C5—C4	−167.3 (5)	C8—C2—C3—N1	2.39 (19)
O6A—N2—C5—C6	11.2 (5)	C8—C2—C3—C4	−177.24 (14)
C6—C1—C2—C3	−2.47 (17)	N1—C3—C4—C5	−178.07 (13)
C6—C1—C2—C8	174.61 (12)	C2—C3—C4—C5	1.6 (2)
C7—C1—C2—C3	176.46 (12)	C3—C4—C5—N2	177.87 (13)
C7—C1—C2—C8	−6.46 (18)	C3—C4—C5—C6	−0.6 (2)
C2—C1—C6—C5	3.43 (19)	N2—C5—C6—C1	179.73 (12)
C7—C1—C6—C5	−175.58 (12)	C4—C5—C6—C1	−1.8 (2)

### Hydrogen-bond geometry (Å, °)

**Table:**

*D*—H···*A*	*D*—H	H···*A*	*D*···*A*	*D*—H···*A*
O1—H1···O2^i^	0.83 (4)	1.80 (4)	2.6216 (16)	175 (3)
C8—H8B···O5A^ii^	0.96	2.55	3.385 (11)	145
C8—H8C···O3A	0.96	2.43	3.023 (11)	120
C8—H8A···Cg1^iii^	0.96	2.96	3.781 (2)	144
C8—H8E···Cg1^iii^	0.96	2.96	3.781 (2)	144

Symmetry codes: (i) −*x*, −*y*, −*z*+1; (ii) *x*, −*y*+1, *z*+1/2; (iii) *x*, *y*−1, *z*.
